# Enhanced hot electron lifetimes in quantum wells with inhibited phonon coupling

**DOI:** 10.1038/s41598-018-30894-9

**Published:** 2018-08-20

**Authors:** Hamidreza Esmaielpour, Vincent R. Whiteside, Herath P. Piyathilaka, Sangeetha Vijeyaragunathan, Bin Wang, Echo Adcock-Smith, Kenneth P. Roberts, Tetsuya D. Mishima, Michael B. Santos, Alan D. Bristow, Ian R. Sellers

**Affiliations:** 1 Department of Physics and Astronomy, University of Oklahoma, Norman, Oklahoma 73019 USA; 2 Department of Physics & Astronomy, West Virginia University, Morgantown, West Virginia 26501 USA; 3 School of Chemical, Biological and Materials Engineering, University of Oklahoma, Norman, Oklahoma 73019 USA; 4 Department of Chemistry and Biochemistry, University of Tulsa, Tulsa, OK 74104 USA

## Abstract

Hot electrons established by the absorption of high-energy photons typically thermalize on a picosecond time scale in a semiconductor, dissipating energy via various phonon-mediated relaxation pathways. Here it is shown that a strong hot carrier distribution can be produced using a type-II quantum well structure. In such systems it is shown that the dominant hot carrier thermalization process is limited by the radiative recombination lifetime of electrons with reduced wavefunction overlap with holes. It is proposed that the subsequent reabsorption of acoustic and optical phonons is facilitated by a mismatch in phonon dispersions at the InAs-AlAsSb interface and serves to further stabilize hot electrons in this system. This lengthens the time scale for thermalization to nanoseconds and results in a hot electron distribution with a temperature of 490 K for a quantum well structure under steady-state illumination at room temperature.

## Introduction

Recently, there has been renewed interest in the potential to manipulate electron-phonon interactions and therefore control hot carrier thermalization in quantum-engineered structures. Novel approaches that implement hot carrier physics have been considered for several applications including high-efficiency solar cells^1,2^, cryogen-free room temperature NIR photodetectors^3^; as well as, to facilitate a new generation of plasmonic devices^4^. Several bulk materials such as InN^5^, BSb^6^, AlSb^7–9^, and InP^10,11^ have shown inhibited hot-carrier relaxation relative to most other materials. Furthermore, a number of groups have demonstrated quantum wells (QWs) with inhibited hot-carrier relaxation relative to bulk systems, suggesting potential applications for QWs as interesting systems for novel next generation optoelectronics^12–15^.

In polar III-V semiconductors, the dominant relaxation pathway for hot carriers is via coupling to LO phonons by the Fröhlich interaction. These LO phonons subsequently dissipate by transferring their energy to multiple acoustic phonons (the Klemens mechanism)^16^, or through the combination of a low energy transverse optical (TO) phonon and an acoustic phonon, a process known as the Ridley mechanism^17^. Systems that limit the efficiency of electron-phonon channels will reduce the efficiency of carrier relaxation and therefore facilitate the stabilization of a hot carrier distribution. The signatures for non-equilibrium carrier populations include longer hot carrier lifetimes^12^, poor thermal conductivity^9^, and the demonstration of carrier multiplication at room temperature^18^.

The origin of inhibited carrier relaxation demonstrated in low-dimensional systems is related to the reduction of the efficiency of the phonon mediated relaxation channel. Much of the early work on hot carriers has focused on the role of inhibited phonon processes during carrier thermalization^1,17,19,20^. The presence of a so-called phonon bottleneck was invoked to describe slowing of the carrier relaxation under conditions of high excitation density across several material systems^6–9^.

A phonon bottleneck describes the existence of a non-equilibrium phonon distribution, typically with a large imbalance in the density of optical and acoustic phonons (LO ≫ LA) due in part to inhibited dissipation of LO phonons. This leads to the re-absorption of LO phonons by electrons, which facilitates a stable hot carrier distribution^21–23^.

Recently, it was proposed that type-II QWs have the potential to inhibit hot-carrier relaxation much more strongly than conventional type-I QWs^24,25^. This mechanism was also observed in silicon colloidal quantum dots^26^, where perturbed hot-carrier relaxation was attributed to a decoupling of the phonons through the spatial separation of photogenerated carriers. In silicon colloidal quantum dots, the presence of hot carriers sustained (even) at low excitations levels was attributed to the indirect band gap of silicon, which served to increase the radiative recombination lifetime of photogenerated carriers. Similar effects have also recently been observed in InAs/AlAsSb QWs^25^.

This manuscript quantitatively investigates the lifetime of hot electrons in InAs/AlAs_0.16_Sb_0.84_ QWs and elucidates the nature of hot-phonon generation and reabsorption processes in these systems. Using continuous-wave photoluminescence (PL), time-resolved terahertz (TR-THz) spectroscopy, and density functional theory (DFT) calculations, it is shown that the combination of long-lived hot electrons and inhibited phonon dissipation appears to result in a strong phonon bottleneck^27^. Moreover, these data suggest that hot carrier generation in type-II QWs is more robust than in type-I systems^20,26^.

## Experimental Results and Analysis

The InAs/AlAsSb QW system has several interesting features that enable hot-carrier effects including: large electron confinement in the InAs QW, due to the conduction band offset of the AlAs_0.16_Sb_0.84_ barrier (Γ (direct) = 1.94 eV; *X* (indirect) ~1.49 eV), and a small confinement for holes in the AlAsSb valence band (offset of only 63 meV)^28^. A further property that makes this system interesting for applications in optoelectronics is the tunable, by varying the QW width, direct InAs band gap. This serves to facilitate strong absorption and generation of photogenerated carriers prior to the rapid spatial separation of carriers as a result of the holes relaxing into the AlAs_*x*_ Sb_1−*x*_ barrier material. This mechanism significantly reduces recombination losses in the QWs and increases the lifetime of photogenerated electrons.

The structure under investigation consists of a 10 nm AlAs_0.16_Sb_0.84_ barrier followed by 30 repetitions of a 2.4 nm InAs QW and a 10 nm AlAs_0.16_Sb_0.84_ barrier grown by molecular beam epitaxy (MBE) on a semi-insulating (SI) GaAs (001) substrate. Prior to the growth of the active multi-QW (MQW) region, the GaAs substrate temperature was increased to 580 °C to remove the native oxide. After the substrate temperature was lowered to 465 °C, a 2 *μ*m thick InAs buffer layer was grown. This layer was used to relax the strain induced by the lattice mismatch at the GaAs/InAs interface; thus, ensuring a nearly fully relaxed lattice-matched template for the rest of the structure. The InAs buffer layer was followed by deposition of the MQW system at 475 °C. Finally, a 50 nm InAs cap layer, to isolate the Al-containing barrier, was deposited at a substrate temperature of 465 °C.

**Figure 1:**
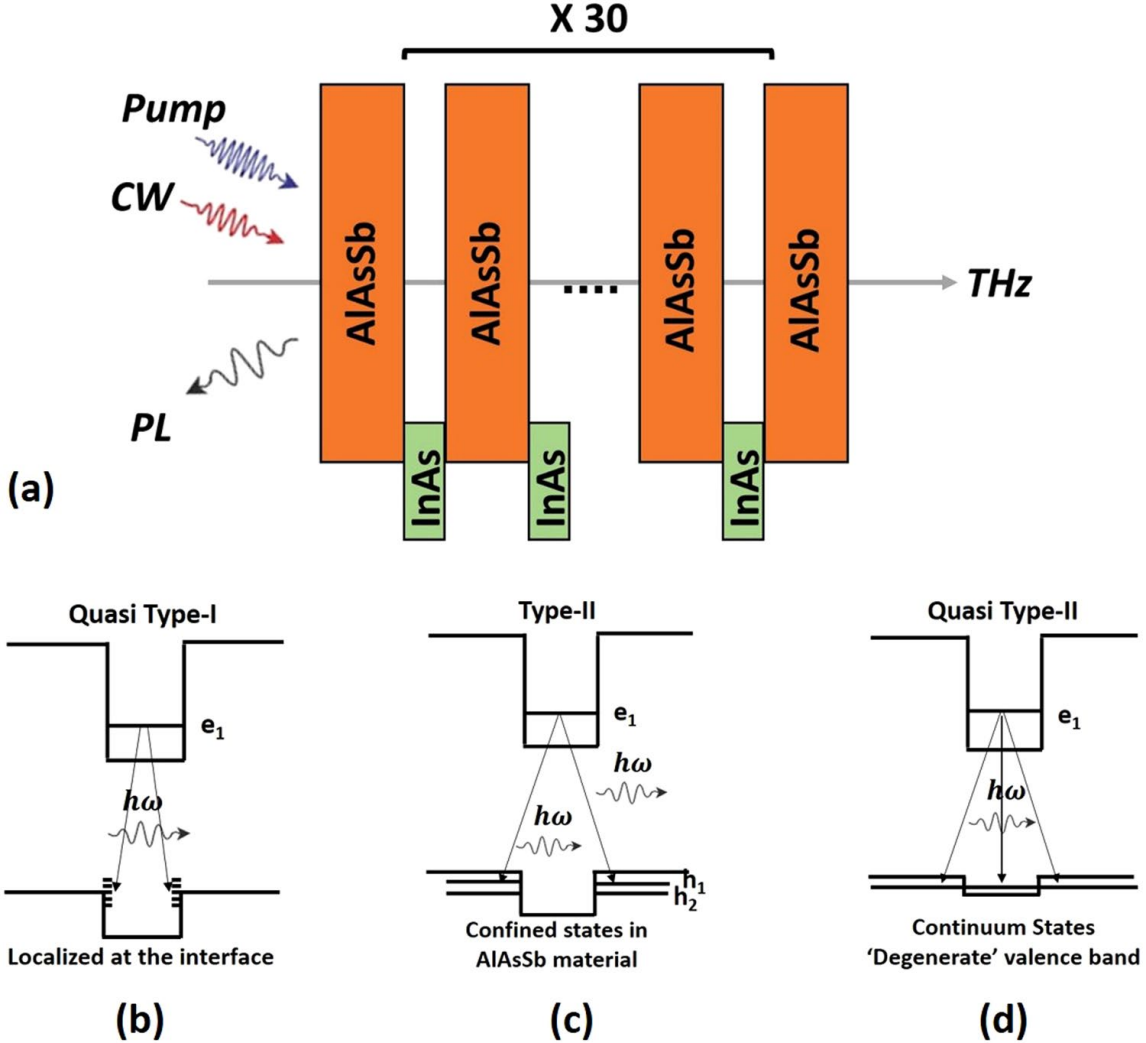
(**a**) Schematic of experimental geometry illustrating the orientation of the pulsed pump and THz probe, as well as, the CW excitation and PL emission of the InAs/AlAsSb MQW structure. (**b**) Quasi-type-I recombination at T < 100 K dominated by quasi direct transitions between holes localized by interface inhomogeneities and electrons in the QW. (**c**) Pure Type-II transitions between electron in QW and holes in the barrier. (**d**) The quasi-type-II situation in which transitions to the less-confined and continuum states for holes dominate at high temperatures.

A schematic of the active region of this structure indicating the geometry and details of the steady state and temporal photoluminescence measurements is shown in Fig. 1(a). Figure 1(b–d) show the *effective* confinement of carriers for the band offsets of the type-II InAs/AlAs_0.16_Sb_0.84_ QWs at different temperatures. Although this system has type-II band offsets, several peculiarities change the nature of the electron-hole interaction strength at different lattice temperatures.

At low temperatures (T < 100), localized states can contribute significantly to the PL of the multi-quantum well structure, as described in our earlier work^24^. The precise nature of the localized states is not known, but we suspect that inhomogeneities near the InAs/AlAs_0.16_Sb_0.84_ interfaces are responsible. For example, the composition of AlAs_0.16_Sb_0.84_, a random alloy, is not perfectly homogeneous due to statistical fluctuations or possibly physical effects such as Group-V diffusion across the interface. In other words, the value of x in the AlAs_*x*_Sb_1−*x*_ layer will vary slightly along the interface. If x is larger than 0.16 for a sufficiently large area along the interface, a wide shallow potential well for holes is formed. Holes can be localized to this area at low temperatures. This interface inhomogeneity would have a negligible effect on electron confinement because the confinement potential for electrons is much larger than for holes.

The localization of holes at the QW interface increases the spatial overlap of the electron and hole wavefunctions at low temperature, which increases the radiative recombination efficiency (producing strong PL) at temperatures below T ~ 100 K. The emission process at low temperature is illustrated schematically in Fig. 1(b) and labeled as quasi-type-I to emphasize the large spatial overlap. As the temperature is increased above 100 K, the hole subbands in the AlAs_*x*_Sb_1−*x*_ layers become significantly populated. The PL is dominated by the type-II band alignment of the QWs, as shown in Fig. 1(c). The spatial separation of carriers results in a rapid decrease in the radiative efficiency of the PL and a subsequent increase in the radiative lifetime of the photogenerated electrons^24,29^.

At higher temperatures, the shallow valence band offset (63 meV) results in the high occupation of continuum states, which strengthens the electron-hole wavefunction overlap. This behavior is illustrated in Fig. 1(d) and labeled as quasi-type-II to distinguish it from the more conventional type-II regime at intermediate temperatures (150 K < T < 225 K).

**Figure 2:**
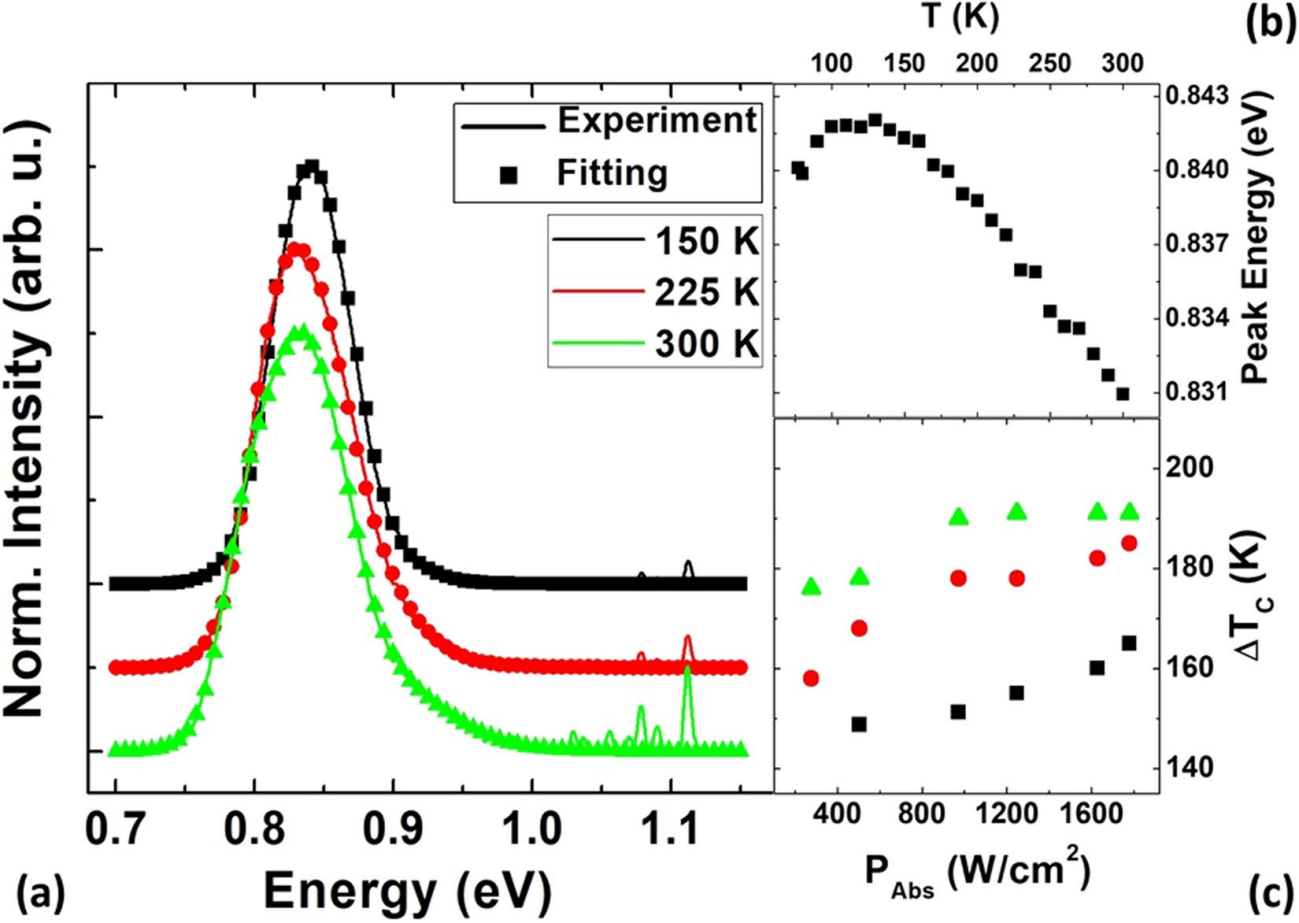
(**a**) Normalized temperature dependent photoluminescence from 77 K to 300 K. (**b**) The peak energy as a function of temperature. (**c**) Extracted carrier temperature differences (Δ*T*_*C*_) as a function of absorbed power at 150 K (black squares), 225 K (red circles), and 300 K (green triangles).

The effect of the band alignment and the relative carrier confinement at various temperatures has been discussed more comprehensively in previous work^24^. The effects are evident in the temperature dependence of the PL shown in Fig. 2(b) (full details of this experiment are described in the Supplementary Information (S1)). Here, the peak energy versus temperature extracted from the PL spectra displays evidence of carrier localization via the presence of an ‘s-shape’ behavior, i.e. a blueshift of the PL energy with increasing temperature. The blueshift and the concurrent reduction in PL intensity with temperature result from the dominance of quasi-type-I transitions (with smaller energy) at the lowest temperature and the increasing importance of type-II transitions (with larger energy) as the temperature is increased.

To concentrate specifically on the type-II regime, the PL analysis is restricted to T > 100 K, where hole localization is limited^24^. In this regime, the long electron lifetime results in hot-carrier stability by inhibiting hot-electron thermalization^24,25^. The normalized PL spectra at T = 150 K (black line), 225 K (red line), and 300 K (green line) are shown in Fig. 2(a). The PL can be modeled via a generalized Planck radiation law^30,31^:

1 $${I}_{PL}(\hslash \omega )=\frac{A(\hslash \omega ){(\hslash \omega )}^{2}}{4{\pi }^{2}{\hslash }^{3}{c}^{2}}{[exp(\frac{\hslash \omega -{\rm{\Delta }}\mu }{{k}_{B}{T}_{C}})-1]}^{-1},$$

where, $\hslash \omega$ is the emitted photon energy, A is the absorptivity, Δ*μ* is the chemical potential or quasi-Fermi-level separation under laser excitation, and T_*C*_ represents the non-equilibrium hot carrier temperature. In the simplest case, taking a linear fit to the high-energy tail of the natural logarithm of a PL spectrum determines the carrier temperature^14,15,24,25,32^. However, such an analysis can be problematic in the case of QWs, where higher carrier excitation (increased laser excitation) and/or increased temperature results in the redistribution and thermal occupation of carriers in the higher energy subbands of the QW^31,33^. These effects serve to broaden the PL in low-dimensional systems at high energy, independent of the carrier temperature.

An advantage of the InAs/AlAs_*x*_Sb_1−*x*_ MQWs investigated here, is that the combination of large energy band offsets and relatively narrow QWs (2.4 nm) leads to a large ground to first excited subband separation (~0.7 eV)^24,25^. This is much greater than the PL linewidth therefore, any excited state broadening due to electrons can be discounted. However, broadening around the PL peak has been observed due to mixing of the valence band states and their contribution to the ground state emission^25^. This becomes apparent at T > 200 K.

To reduce intrinsic broadening of the extracted carrier temperature, the full PL spectrum is fit using Equation (1) and the energy dependence of the absorption (A) is determined by considering the relative position of the quasi-Fermi energy positions for Δ*μ*. Figure 2(a) shows the fit of the PL at full power (6.3 mW) at 150 K (black squares), 225 K (red circles), and 300 K (green triangles). These temperatures were selected because the QWs are dominated by band-to-band radiative recombination rather than via holes localized by interface inhomogeneities^24^. The T > 100 K regime is also where robust hot carrier temperatures have been observed previously^24,25^.

In Fig. 2(c) the effective temperature of the electrons above the lattice (Δ*T*_*C*_ = T_*C*_ − T_*L*_) is shown with increasing absorbed power. It is clear that the hot-carrier temperature is weakly dependent on excitation power, but becomes significantly hotter with increasing lattice temperature. This has been discussed in relation to the increased photogenerated electron lifetime in the QWs, which is correlated to the type-II nature of the system^25,26^.

This high electron temperature and weak power dependence are consistent with earlier work on these structures, where non-equilibrium electron temperatures and insensitivity to excitation powers (at much lower intensities than used here) were evident once holes were delocalized from interface inhomogeneities and the true type-II nature of the system was revealed^24,25^. A consequence of this behavior is that Δ*T*_*C*_ increases with increasing lattice temperature, *T*_*L*_, despite even higher concentrations of optical and acoustic phonons as the temperature increases.

It was hypothesized that shortly after non-equilibrium electron and hole distributions are created by photo absorption in the InAs QWs, the holes rapidly relax into the AlAs_*x*_Sb_1−*x*_ barriers. This spatial separation of electrons from holes increases their radiative lifetimes. It is proposed that this process, coupled with the increasing electron density in the QWs (~10^9^ cm^−2^) with increasing temperature, causes an inhibited electron-phonon relaxation and a hot-carrier distribution in the steady state^26^.

Quantitative carrier lifetimes are determined by TR-THz, which measures the AC photocurrent as a function of delay time between a near-infrared pump pulse (at 1.03 eV with an average power of 4 mW) and a THz probe pulse. To facilitate transmission for the TR-THz measurements, the InAs buffer layer between the GaAs substrate and the active region was removed with a combination of mechanical polishing and a dilute selective wet-etchant. Full details of this process are given in the Supplementary Information (S1). Details of the transient absorption measurements are described in detail elsewhere^34^ and further details are also given in the Supplementary Information (S3).

**Figure 3:**
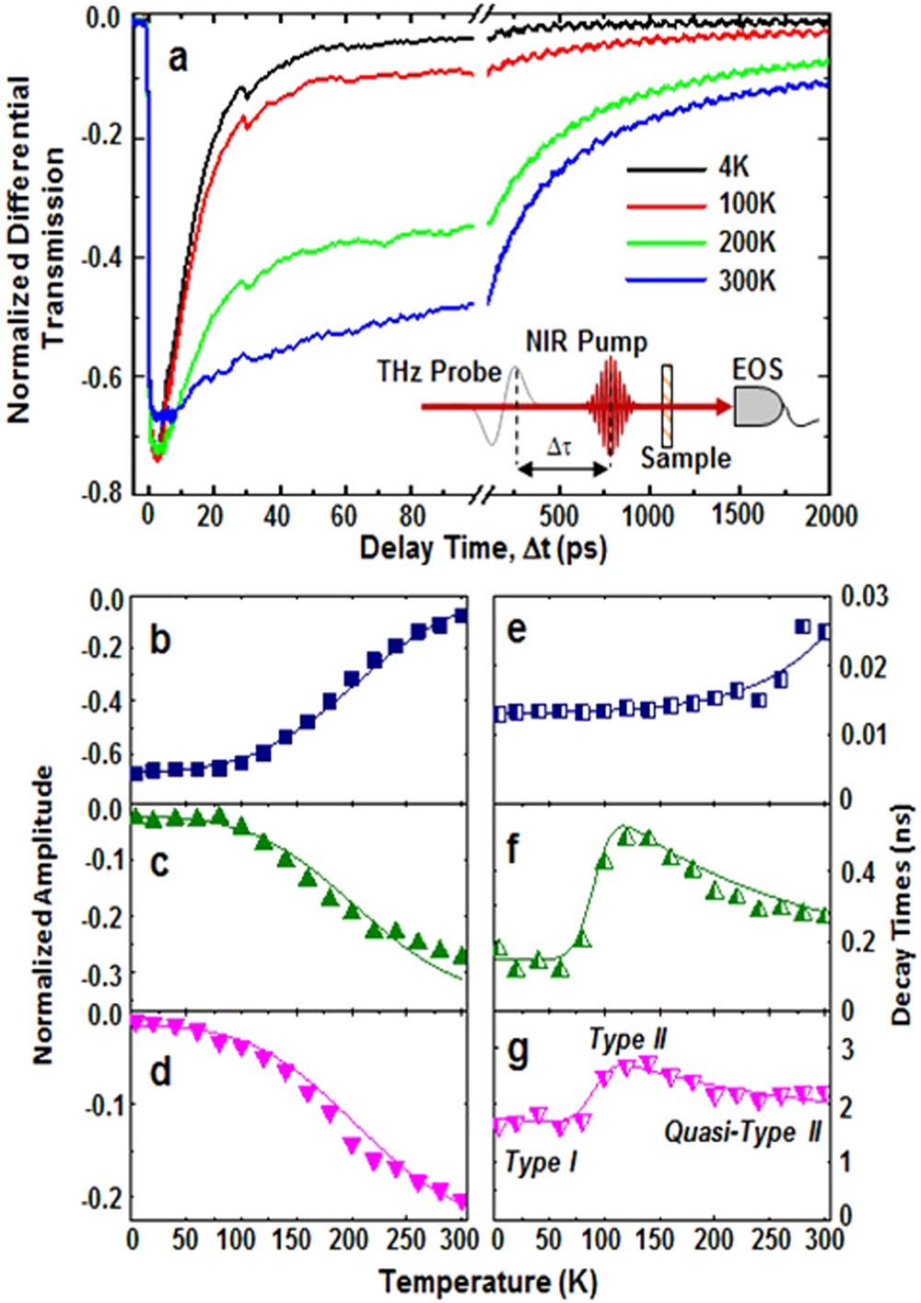
Carrier dynamics of MQW system: (**a**) Normalized differential terahertz (TR-THz) transmission for a range of lattice temperatures. The inset shows the collinear experimental geometry of THz probe and near-infrared (NIR) pump. (**b**–**d**) and (**e**–**g**) are the amplitude and decay times of the fast, intermediate, and slow carrier dynamics extracted from fitting the transients. In (**g**) the regions of quasi-type I, type II, and quasi-type II are labeled.

The measurements presented in Fig. 3(a) are recorded at the maximum THz field and only respond to the change in THz absorption Δ*E*(*t*)/*E* as a function of the pump-probe delay time (t) due to free carriers created by pumping above the band edge of the QWs. These free carriers cause additional absorption which reduces the peak of the THz electric field. Transients are recorded for a range of temperatures from 5 K to 300 K that resolve both fast and slow decay dynamics. The fast decay process, which dominates at low temperature, is followed by slower decay processes that become more dominant at higher temperatures.

The transient decays are best fit by a three-component model, which accounts for recombination through at least two sets of states. This is given by:

2 $${\rm{\Delta }}E(t)/E=\sum _{i=1}^{3}{A}_{i}\exp [\,-\,(t-{t}_{0})/{\tau }_{i}],$$

where *A*_*i*_ and 1/*τ*_*i*_ are the amplitude and decay rates of each component. This neglects the fast rise-time recorded in the measurements, which is on the order of the cross-correlation of the excitation and THz pulses. Consequently, Fig. 3 focuses on the decay processes and plots the temperature dependence of the amplitudes (b–d) and decay times (e–g). Visual inspection of the three decay contributions reveals the fast component (i = 1, Fig. 3(b,e)) to be significantly different mechanism from the intermediate (i = 2, Fig. 3(c,f)) and slow (i = 3, Fig. 3(d,g)) components. First, the amplitude of the fast decay (Fig. 3(b)) decreases with temperature and has a slight monotonic increase in lifetime (Fig. 3(e)) with temperature. The fast component is attributed to the quasi-type-I recombination process between the ground conduction state e1 and the localized hole states arising from interface inhomogeneities as illustrated schematically in Fig. 1(b)^24,25^.

In contrast, the slower components grow in amplitude (Fig. 3(c,d)) with temperature and have distinct non-monotonic lifetimes (Fig. 3(f,g)). This increasing lifetime with temperature directly probes the type-II (Fig. 1(c)) transition and the thermally activated carrier escape above 100 K. Moreover, the temperature dependences of the two slower components are highly correlated, most likely indicating that they represent a two-step decay process between the same set of initial and final states. These competing fast and slower components are attributed to the redistribution of photogenerated holes and the effective degeneracy of the valence band at elevated temperatures; which leads to competition and the convolution of PL from multiple confined hole states and the ground state electrons. Summing the three transient amplitudes reveals near complete transfer of dominance from the fast to the slower components with increasing temperature (see Supplementary Information S3). This means that the transfer in amplitudes is also dominated by the availability of a certain set of states involved in the recombination. Since hole states arising from interface inhomogeneities are preferentially occupied at low temperature, it is their availability with holes as the minority carriers that dominate the temperature dependence of all the allowed transitions.

Modeling these states as an allowed direct transition reveals that they have a 17.5 meV energy difference from the hole states in the barrier with a 9.5 meV spread (see S3), which is equivalent to the activation energy associated with localized to free carrier regime (type-I to type-II) determined previously^24^. The subtle effect of these competing overlapping hole states has been considered more extensively elsewhere^28^. Modeling the fast lifetimes reveals a near temperature independent lifetime of about 13 ps (Fig. 3(e)), with a slight increase at higher temperature that can be attributed to increased uncertainty as the significantly longer-lived decay contributions become dominant. At low temperature, the quasi-type-I recombination (Fig. 1(b)) suppresses the time decay of the slower contributions. However, as the temperature is increased, the type-I-like components are switched off and the true type-II structure dominates the decay times (Fig. 1(c)). At very high temperatures, the holes are primarily in continuum states. The system is *quasi-type-II* (i.e., partly type-I – Fig. 1(d)), which lowers the decay times of the slower carrier dynamics.

The qualitative model starts with the photoexcitation of carriers above the QW band gap, which initially creates a non-thermal distribution of photogenerated carriers. This evolves into a Boltzmann distribution on a femtosecond timescale through electron-electron interactions. This process is significantly faster than the electron-phonon interaction that only starts to thermalize the system after a picosecond. The electrons begin to thermalize via Fröhlich interactions resulting in the emission of LO phonons. Thereafter, these LO phonons decay into acoustic phonons either via the Ridley^17,22^ or Klemens process^16,22^. The dissipation of heat is then completed by the lateral propagation of the thermal energy via thermal conductivity. Since the majority of the excess energy is transferred to the electron population, the holes are considered thermalized^19,35^. Confirmation of the long-lived nature of the photogenerated electrons in the InAs QWs supports the notion that the carriers in the QW facilitate a phonon bottleneck.

**Figure 4:**
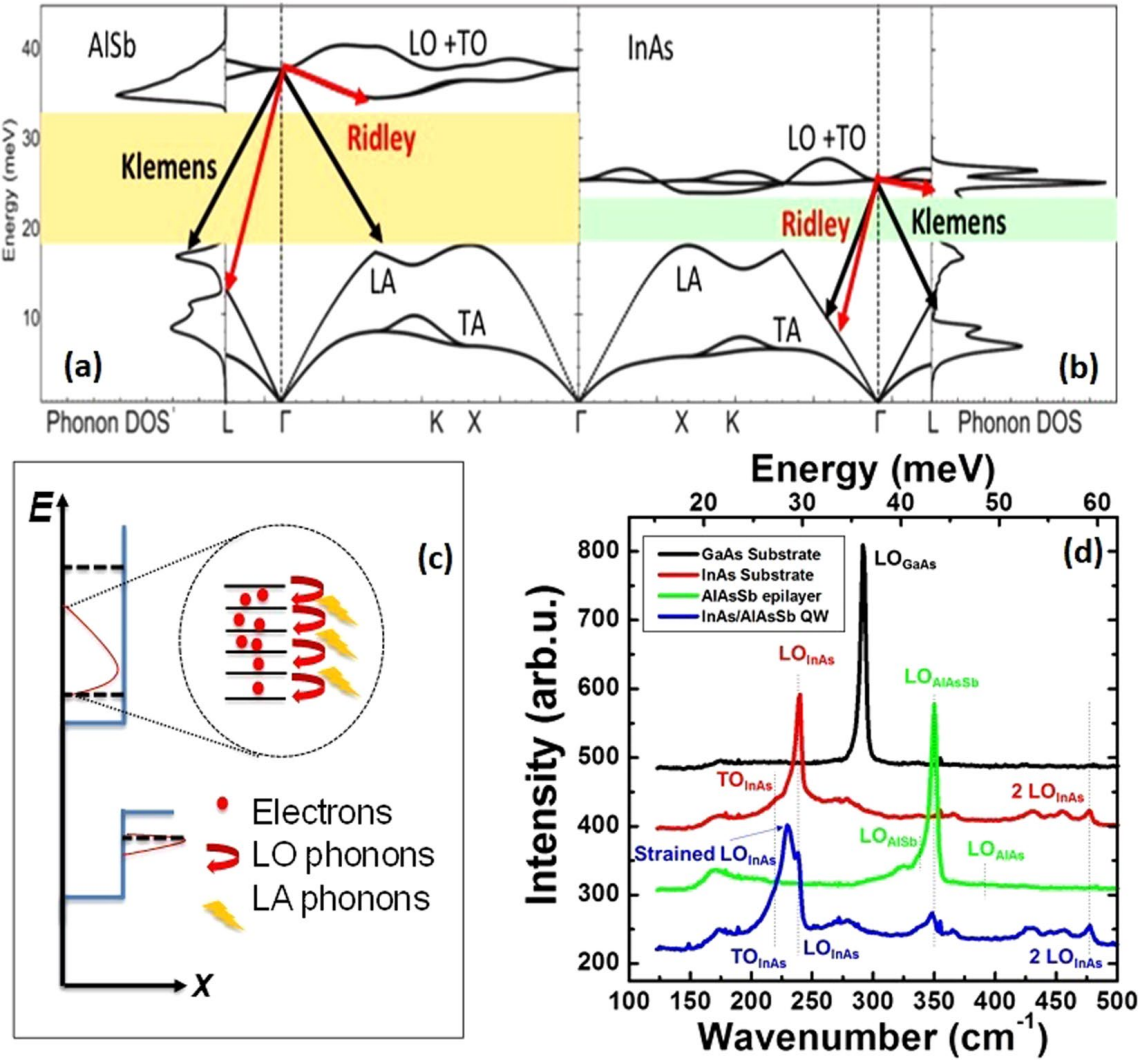
Phonon density of states and dispersion calculated using DFT analysis for (**a**) AlSb and (**b**) InAs. The shaded regions indicate the phonon band gap. Also shown are examples of the Klemens (black) and Ridley (red) relaxation channels. (**c**) The magnified image of the hot electron distribution (full red circles) shown in the dotted circle illustrates the relaxation of carriers through LO phonon emission (solid red arrows) and the subsequent generation of LA phonons (solid yellow bolts). (**d**) Raman spectrum of GaAs (black), InAs (red), and AlAs_0.16_Sb_0.84_ (green) reference samples, and the full InAs/AlAs_0.16_Sb_0.84_ multi-quantum-well structure (blue).

A schematic of this basic thermalization pathway is illustrated in Fig. 4(c), which shows the non-equilibrium photogenerated electron distribution (brown line) in the ground subband. The carrier thermalization process proposed is illustrated by the magnified region in Fig. 4(c). This schematic shows several hot electrons (solid red circles) that each subsequently emit an LO phonon (full red arrows) that decays into LA phonons (yellow bolts). At high optical excitation (and therefore high carrier density), LO phonons are generated via the Fröhlich interaction at a faster rate than the Klemens and Ridley interactions can convert the LO phonons into acoustic phonons. This phonon bottleneck enables the LO phonons to be reabsorbed by electrons, which helps to sustain the hot-electron population. This hypothesis is investigated here by implementing a more focused analysis on the role of phonons in the stabilization of hot carriers.

Figure 4(a,b) show the phonon dispersion across a single interface of the system as predicted by first-principles DFT calculations using the VASP package^36^. Figure 4(a) shows the AlSb phonon density of states and dispersion while Fig. 4(b) shows the same information for InAs. Here, AlSb is used to simplify the calculations for the modeling of the barrier material. This is justified since the As-composition in the experimental structure is low (16%), and therefore qualitatively similar to AlSb. It is assumed that the basic properties of the bulk provide a reasonable first order description of the behavior of the MQW system.

On inspection, the phononic properties exhibit a large phonon energy offset between the InAs QW and the AlSb barrier model material at the Γ-point, which is where radiative recombination and LO phonon emission/absorption take place. InAs has a relatively small phonon band gap (optical to acoustic phonon energy gap indicated by the light green, shaded region in Fig. 4(b)), and an optical phonon (LO) energy of ~29 meV in the bulk. Although the asymmetric Ridley mechanism (red arrows)^17^ is not significant in InAs due to the strong mixing of the LO and TO modes in this system, this is not the case for the Klemens decay channel.

The Klemens mechanism (black arrows in Fig. 4) specifically involves the decay of hot LO phonons into two energetically equivalent LA phonons whose momenta are equal in magnitude but opposite in direction. The Klemens channel is inhibited if the minimum optical phonon mode ($\hslash {\omega }_{LO}$) is two times larger than that of the energy of the highest LA phonon ($\hslash {\omega }_{LA}$) branch (i.e. $\hslash {\omega }_{LO}/\hslash {\omega }_{LA} > 2$); which is typically seen in compounds with a large difference in the atomic mass of the constituent atoms. For InAs this ratio is small ($\hslash {\omega }_{LO}/\hslash {\omega }_{LA}\sim 1$), indicating the importance of the Klemens pathway, which dominates and determines the carrier thermalization rate for bulk InAs^16^.

In the case of AlSb there is a large difference in the cation and anion mass, resulting in a larger phonon band gap (tan shaded region of Fig. 4(a))^8,16^ than for InAs. Since the ratio of the optical and acoustic modes is ~2 for AlSb, the Klemens path is significantly reduced. Although the Ridley channel is much less significant in AlSb^8,17^ it is also non-negligible^7,8^. The combination of inhibited Klemens channels plus a non-negligible Ridley component, make AlSb an interesting material for hot carrier applications.

Despite its promise, AlSb is a high-energy indirect semiconductor (as is AlAs_0.16_Sb_0.84_), and its applicability to optoelectronics is therefore likely limited. This material’s ability to inhibit the propagation of heat away from the active region (QW) however, suggests that it is a useful barrier material for hot carrier systems. A consequence of the large atomic mass difference in this material is to severely restrict the optical-acoustic scattering processes, which accounts for the low thermal conductivity observed in AlSb^9^.

This, coupled with the phonon energy mismatch at the InAs/AlSb interface (see Fig. 4), further inhibits the lateral propagation of acoustic phonons and dissipation of heat away from the QW region. Even though LO phonons couple relatively efficiently into acoustic phonons in the InAs QW, the propagation of these LA modes are inhibited at the AlSb interface. While inhomogeneities at the interface^35^ likely support some propagation, or leakage, of surface modes across the interface into the barrier from the QWs, the net result of these properties is that the majority of acoustic phonons are reabsorbed (and/or reflected) back into the QW.

Interestingly, the Klemens mechanism, which dissipates hot LO phonons in this system, also appears to facilitate the reabsorption of LA phonons – if their lateral dissipation is inhibited^17,35^. This reabsorption of LA phonons leads to the generation of additional LO phonons (reverse of Klemens process) and a *stronger bottleneck*. Such behavior has also been invoked recently in the perovskite systems^37^, where the weak dissipation of LA phonons was attributed to the poor thermal conductivity of the inorganic linkers that stabilize the molecules in these systems^38^. Once reabsorbed, the up-converted LA phonons effectively couple to LO phonons to further increase the hot LO phonon density; thus, re-heating and stabilizing the hot electron distribution in the InAs QW.

Although the physical picture proposed requires further quantitative investigation of the LO phonon lifetime and acoustic phonon characteristics, the experimental and initial theoretical data presented are wholly consistent with the processes described. The net result is the generation of a large population of LO phonons in the InAs/AlAsSb QWs investigated, which is enhanced in the active region by the poor heat transport of AlAsSb. Moreover, evidence of this type of acoustic phonon bottleneck has also been reported in GaAs and to a much larger extent in bulk CdSe^39^. In the case of CdSe, it is thought to be facilitated by the strong anharmonic coupling between acoustic and optical phonons in polar semiconductors^11^.

Confirmation of the large difference in optical phonon modes are shown in Fig. 4(d), which presents a comparison of the Raman spectrum (details are in S4) for the InAs/AlAsSb MQW structure (blue) with respect to an AlAs_*x*_Sb_1−*x*_ epilayer (green) and bulk reference substrates of InAs (red) and GaAs (black); all of which are incorporated in the full MQW structure. The large difference between the InAs and AlSb optical phonon energies predicted in Fig. 4(a,b) are clearly reflected in the Raman data from the AlAs_*x*_Sb_1−*x*_ and InAs layers; signatures of which are also present in the Raman data of the full InAs/AlAs_*x*_Sb_1−*x*_ structure (blue). The contribution of Raman peaks due to the AlAs_0.16_Sb_0.84_ barrier is relatively weak in the full MQW structure, which is dominated by the LO phonon contribution of the strained InAs buffer (230 cm^−1^) and InAs QWs (238 cm^−1^). This is further evidence of both the limited contribution of phonon processes in the barriers in carrier thermalization and the strong confinement of hot electron-phonon processes within the QWs.

The cooling processes in the QW were also modeled using an energy loss process in which the LO-phonon emission was invoked to determine the dynamics of the system (see Supplementary Information S7). This analysis also supports the creation of a stable and non-equilibrium coherent hot LO phonon reservoir due to the large and long-lived carrier density photogenerated by CW excitation in the QWs. This behavior facilitates the creation of a stable non-equilibrium hot electron distribution in the QWs, with LO phonon lifetimes on the order of several picoseconds. This is significantly longer than the lifetime seen in III-V systems, which is typically on the order of femtoseconds^37^.

A more detailed description of the phononic properties of the coupled InAs-AlAsSb system, including effects related to relaxation of momentum conservation at the interfaces, zone-folding, phononic confinement in the superlattice, and the effects of thermal transport across the interfaces is now underway.

## Conclusions

In conclusion, it is proposed that the experimental observation of robust hot electrons in InAs/AlAs_*x*_Sb_1−*x*_ quantum wells at T > 100 K is the combined result of long-lived photogenerated carriers in the type-II QWs and inhibited LO phonon dissipation from the active region. The net effect is an acoustic phonon bottleneck and the subsequent re-absorption of LO phonons by electrons. This results in a stable hot-electron distribution in the QWs.

## Electronic supplementary material

Supplementary Information
