# Supplementary material for: Enhanced hot electron lifetimes in quantum wells with inhibited phonon coupling
Source: Sci Rep. 2018 Aug 20;8:12473. doi: 10.1038/s41598-018-30894-9 (PMC6102289; doi:10.1038/s41598-018-30894-9)
Supplement: Supplementary file 1 — Supplementary Information [file 41598_2018_30894_MOESM1_ESM.pdf]

Supplementary Information *for*

Enhanced hot electron lifetimes in quantum wells with inhibited phonon coupling

H. Esmaielpour[1], V. R. Whiteside[1], H. P. Piyathilaka[2], S. Vijeyaragunathan[1], B. Wang[3], E. Adcock-Smith[4], K. P. Roberts[4], T. D. Mishima[1], M. B. Santos[1], A. D. Bristow[2], and I. R. Sellers[1*]

[1] *Department of Physics and Astronomy, University of Oklahoma, Norman, Oklahoma, 73019, USA*
[2] *Department of Physics & Astronomy, West Virginia University, Morgantown, West Virginia, 26501, USA*
[3] *School of Chemical, Biological and Materials Engineering, University of Oklahoma, Norman, Oklahoma, 73019, USA*
[4] *Department of Chemistry and Biochemistry, University of Tulsa, Tulsa, OK 74104, USA*
** Corresponding author: Ian R. Sellers, email: sellers@ou.edu*

S1: Sample processing: First, the 50 nm InAs cap layer was removed by immersing the sample into a selective wet echant (concentrated citric acid (1gr citric acid powder:1mL DI water):H_2O_2 with (5:1) concentration) for 60 sec. Then, the sample was mounted upon a transparent sapphire substrate using an ultraviolet adhesive (NOA88) to remove the large portion of the GaAs substrate via mechanical polishing. The remaining part of the substrate (15 µm) was removed using a selective wet chemical etchant (NH_4OH:H_2O_2:H_2O with (1:3:16) concentration) with a etch rate of 1.5 µm/min. Finally, the sample was immersed in the same solution as the one for the InAs cap layer for 40 min to remove the whole InAs buffer layer (2000 nm). At the end, the outcome is a submicron layer of only InAs/$AlAs_{1-x}Sb_x$ MQW active region attached to the sapphire. A schematic of the sample geometry upon completion of the post-growth processing is shown as an inset in Figure **S1.1**.

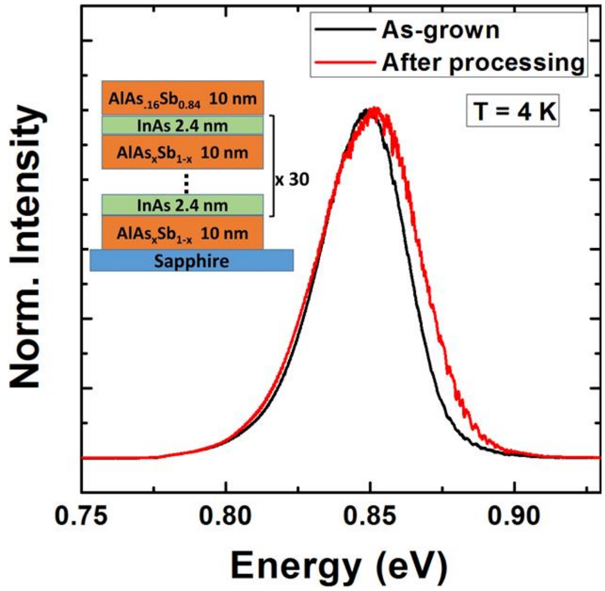

Figure S1.1: Normalized photoluminescence spectra before (black) and after (red) processing. The small blueshift in the PL spectrum after processing is indicative of strain relaxation of the InAs QW after removing the GaAs substrate and InAs buffer layer. Inset: Schematic illustration of multi-quantum-well structure mounted on sapphire using an ultraviolet curing adhesive (NOA88) after removal of InAs cap, InAs buffer layer, and GaAs substrate.

A comparison of the PL before and after processing is shown in Figure **S1.1**, measured at 4.2 K. Comparing the PL before (black) and after (red) removal of the substrate shows a blueshift in the PL peak energy, which indicates some

strain relaxation and narrowing of the lattice constant upon removal of the GaAs substrate and InAs buffer region. This most likely is due to dislocations in the InAs buffer layer, indicating the active region experienced a small contribution of tensile strain prior to removal.

S2: CW Photoluminescence measurements were performed using a confocal micro-photoluminescence system with a spot size of ~ 5.66 μm and excitation wavelength of 632.8 nm. The luminescence was then dispersed through a Spex 270M spectrometer and detected using a LN_2-cooled single-channel germanium detector. The total absorbed power was determined using a transfer matrix calculation that includes the optical constants of the system, a maximum incident power of 6.38 mW over an excitation area of 5.66 μm, and an optical excitation at 632.8 nm to determine the energy dependent reflectivity and absorption profiles. These parameters were carefully extracted using the pinhole method in combination with a calibrated optical power meter and optical imaging of the emitted PL luminescence region of the sample using a NICAM digital camera.

S3: Time-Domain Terahertz measurements: Details of the transient absorption measurements are given in detail elsewhere [Senty et al]. In brief, ~100 fs pulses are created by a 1 kHz regenerative laser amplifier at 800 nm and an optical parametric amplifier (OPA), tunable through the near infrared. THz is generated by optical rectification of 800-nm pulses in a 1-mm thick (110) $CdSiP_2$ crystal. THz pulses transmit through the sample in the cryostat, are recollected and detected using electro-optic sampling in a 1-mm thick (100) ZnTe crystal by a 800-nm gate pulse, which probes the electric field of the THz directly. In the time-domain, varying the delay time between the THz and gate pulses arriving at the electro-optic crystal (t_{THz}) maps the entire pulse shape of the THz electric field, and hence its frequency components.

An optical pump beam is sufficiently strong to excite a photoexcite carrier with a density of ~10^{14} cm^{-2} per well based on the absorbance at 1 eV, the number of wells and the average power of the laser beam at the sample. For pump-probe measurement t_{THz} are set to the maximum of the THz transient to make the signal resulting from optical pumping is dominated by free carrier absorption. Then the delay time ($\Delta\tau$) between the THz probe and the optical pump can be varied over a range of 2 ns, with < 1 ps time resolution. The optical pump is mechanically chopped at a frequency that is a subharmonic of the laser amplifier and the resulting differential transmission is detected in a lock-in amplifier, referenced to the pump modulation frequency.

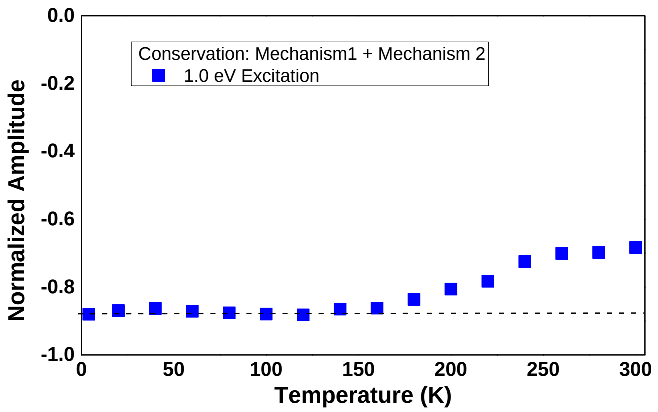

Figure S3.1: Temperature dependence of the sum of the amplitudes extracted from the transient absorption at each temperature. Mechanism 1 corresponds to A_1, mechanism 2 corresponds to A_2+A_3.

Amplitudes and decay components are extracted from the transient absorption measurements as a function of temperature. These results are presented in the Figure 3 of the paper. Examining the temperature dependences of the amplitudes indicated that as the fast component, which dominates at low temperature, gives way to the slower two components as the temperature is increased there seems to be some equality to the two processes. This is further illustrated by summing A_1, A_2 and A_3 and plotting the result as a function of temperature; see Figure S2. As temperatures below 200 K there is a total amplitude of signal of -0.8 %, which indicates a near complete conversion of decay mechanism 1 (the fast component) to decay mechanism 2 (the intermediate and slow components). This only starts to decay at higher temperatures indicating another process that reduces the absorption of pump light into a free-carrier absorption signal that modifies the transmitted THz radiation.

To fit the temperature dependence a qualitative model is used to links the availability of the alloy fluctuation states with the amplitudes of the decay mechanisms. In addition, the lifetimes of those states are associated with faster or slower dynamics. First, it is assumed that at low temperature recombination fast recombination can occur through the direct recombination between electron states and alloy fluctuation states within the well (quasi-type I system). The amplitude is determined as:

$$A_1(T) = \frac{A_1(0)}{2} \mathrm{erfc}\left[\frac{T-\Delta E_{dh}/k_B}{w_{dh}/k_B}\right], \quad \text{(S1)}$$

where $A_1(0)$ is the initial amplitude at low temperature, T is the temperature, $\Delta E_{dh}/k_B$ is the temperature where alloy fluctuation states become unavailable and w_{dh}/k_B is the range of temperature from completely available to unavailable; hence, ΔE_{dh} and w_{dh} are the corresponding energies of those last two variables.

Figure S2 shows that as complete conversion between the fast direct and the slower indirect decay mechanisms is nearly valid. Hence, the higher temperature reduction of the fast amplitude results in a growth of the slower indirect recombination mechanisms. This occurs between electrons in the well and holes in the barrier. Since both intermediate and slow recombination has nearly identical temperature dependences, the inverse expression to Equation (1) describes their behaviour:

$$A_{2,3}(T) = A_{2,3}(0) + C_{2,3}\frac{A_1(0)}{2}\left(1 + \mathrm{erf}\left[\frac{T-\Delta E_{dh}/k_B}{w_{dh}/k_B}\right]\right), \quad \text{(S2)}$$

where $A_{2,3}(0)$ is the low temperature decay rate for the intermediate (2) and slow (3) decay contributions which is not forbidden only less likely, and $C_{2,3}$ are branching ratios for the two decay contributions as they become more likely at higher temperatures.

Overall in Equations (1) and (2), the only relevant parameters for understanding the energy scales involved in the recombination amplitudes are ΔE_{dh} and w_{dh}. Their values are given in the main paper and agree well with the peak energy shift and broadening of the PL data. All other parameters are fitting parameters associated with the total available densities of states of this specific sample.

Fitting the decay times for the fast (1), intermediate (2) and slow (3) contributions requires different behaviour for the fast component in comparison to the two slower components. There also appears to be interplay between the three. At low temperature, the fast component dominates and suppresses the decay time of the slower components. At intermediate temperature the sample transitions from quasi-type-I to type-II, which increases the time constants of the slower components, and finally at higher temperature the delocalization of the hole states throughout the entire quasi-type-II system reduces the slower time constants while increase the faster one.

The fast decay time is modelled as a low temperature component, $\tau_1(0)$ = 13 ps, with an increasing temperature dependent contribution, τ_{1T} = 0.08, such that:

$$\tau_1(T) = \tau_1(0)[1 + \tau_{1T}\exp(k_BT/\Delta)], \quad \text{(S3)}$$

where Δ = 5.5 meV is the increase factor with temperature.

The intermediate and slower decay contributions are given by:

$$\tau_{2,3}(T) = \tau_{2,3}(0)\left[1 + \tau_{2,3T}\left(1 + \mathrm{erf}\left[\frac{T-T_T}{T_w}\right]\right)\exp\left(-\frac{k_BT}{\Delta_{HT}}\right)\right], \quad \text{(S4)}$$

where $\tau_2(0)$ = 150 ps and $\tau_3(0)$ = 1700 ps are fitting parameters for the suppressed low temperature time constants respectively, τ_{2T} = 2.6 and τ_{3T} = 0.6 are the rates of increase when the system becomes type-II. In both cases, the intermediate behavior is dominated by the second-order transition with temperature (T_T = 95 k) and transition width (T_w = 20 K) and finally by the reduction of these indirect transition times by Δ_{HT} = 14.6 meV.

It should be noted this method is more challenging for interpretation because there is clear interplay between the observed decay time. An alternative method would be to sample the data and differentiate to obtain (dN/dt), then plot it against the extracted excited carrier concentration from the absorbed power to obtain N. This is the rate-equation and shows how at different excited carrier concentrations the rate is dominated by clear and separable mechanisms.

S4: Raman Spectroscopy: Raman spectroscopy was performed using an Witec (Ulm, Germany) spectroscopy system equipped with microprobe Raman capabilities. A 514 nm Melles Griot 43 argon ion laser (Carlsbad, CA) was fiber-coupled to the instrument at a power of 20 mW. A typical 1800 g/mm grating was used with a 20x focusing objective and an exposure time of 30 seconds with an 8x8 hardware and software binning. Samples were placed on an x,y translation stage and probed at several points to ensure sample homogeneity. Data were collected using a thermoelectrically-cooled CCD camera, cooled to –60°C.

S5: Simulated band alignment and conduction and valence band energy band offsets: As shown in figure S5.1, the energy separation in the quantum well (conduction band) is large (=700 meV), however, there are multiple energy levels in the valance band with small separation which makes the state filling effect be feasible in the system. The indepth discussion about the state filing effect due to the hole levels is described elsewhere. [1]

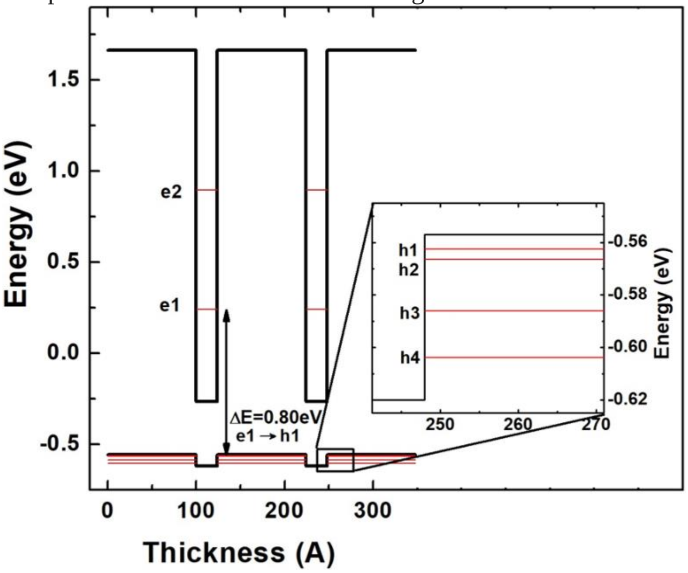

Figure S5.1: Schematic of band energy diagram of the InAs QW (24 A) with AlAsSb barrier (100A). The inset shows the magnified region of valence band with multiple energy levels with small separation.

S6: Density Functional Theory Calculations: DFT Calculation were performed using VASP package [2]. The PBE-GGA exchange-correlation potential [3] was used and electron-core interactions were treated in the projector augmented wave (PAW) method [4,5]. The Brillouin zone was sampled with a k-point (11 × 11 × 11) for the InAs and AlSb unit cells for the structural optimization. During the optimization, all the atoms were free to relax until the self-consistent forces reached 0.02 eV / Å. The phonon calculations were carried out by calculating force constants in real space using the Density Functional Perturbation Theory (DFPT) [6, 7].

S7: LO Phonon scattering lifetime: To further quantify the effect of phonon localization and scattering in the QW, the following equation for phonon emission and reabsorption in the hot phonon regime is considered: [8,9,10]

$$P_{th} = \frac{\hbar\omega_{LO}}{\tau_{eff} m}\left(exp\left(-\frac{\hbar\omega_{LO}}{k_B T_L}\right) - exp\left(-\frac{\hbar\omega_{LO}}{k_B T_C}\right)\right), \qquad \text{(S5)}$$

where p_{th} is the thermalized power, E_{LO} is the optical phonon energy in the InAs QW (29 meV), τ_{eff} describes the *effective* lifetime of the hot LO phonon distribution, k_B is Boltzmann's constant, T_C is the electron temperature, and T_L is the equilibrium lattice temperature.

The thermalized power, p_{th}, represents the fraction of absorbed power that is transferred to the lattice in terms of heat generation through lattice-vibrations (phonons). p_{th} is equivalent to the absorbed optical power, P_{abs} [8,11] since there is no electrical connection to the sample and most of the photons generated by absorption of the laser excitation suffer total internal reflection - due to the large refractive index contrast at the semiconductor/air interface.

Figure S7.1 shows the inverse T_C versus T_L plot for temperatures ranging from 77 K to 300 K. T_C is extracted from the PL using Equation (S5), the fits to determine T_C are shown in Figure S7.1 (solid symbols) for several lattice

temperatures (T_L). These measurements were performed at three different powers: low (1250 W/cm^2 – green triangles); medium (1630 W/cm^2 – red circles); and high (1780 W/cm^2 - black squares). These absorbed powers translate to photogenerated carrier densities of n = 4.0x10^{12} cm^{-2}, 5.2x10^{12} cm^{-2}, and 5.7x10^{12} cm^{-2}, respectively; as determined from transfer matrix (TM) calculations of the structure based on a recombination lifetime of 1 *ns*, which is consistent with the lifetime of the carriers extracted in the THz-TDS measurements described above.

In Figure S7.1, T_C is also included for PL at T_L < 150 K despite the effects of carrier localization at these temperatures. This does not significantly change the discussion since the effects of carrier localization are limited at the relatively high absorbed power densities presented; this is due to saturation of the localized states at high photogenerated carrier densities, [11,12] even at lower temperatures. The fact that these lower temperature PL data do not significantly change the slope of the T_C versus T_L response observed in Figure S7.1 further supports inclusion of the T_L < 150 K data.

The inset to Figure S7.1 shows an alternative to the T_C versus T_L plot, specifically taking Equation (S5) and plotting the exponential components of T_C versus that of T_L. This provides a method to find the effective lifetime of the phonon distribution, τ_{eff}, and therefore, the stability of hot electrons in the system. The dotted lines shown on the data in the inset to Figure S7.1 show the fit to the experimental data with τ_{eff} *extracted* for this system using Equation (S5). From this fit an effective lifetime of 1.9 *ps* is experimentally determined. τ_{eff} represents to scattering rate, or lifetime of the LO phonons in the InAs QW, which is an order of magnitude higher than that typically determined in bulk III-V's.

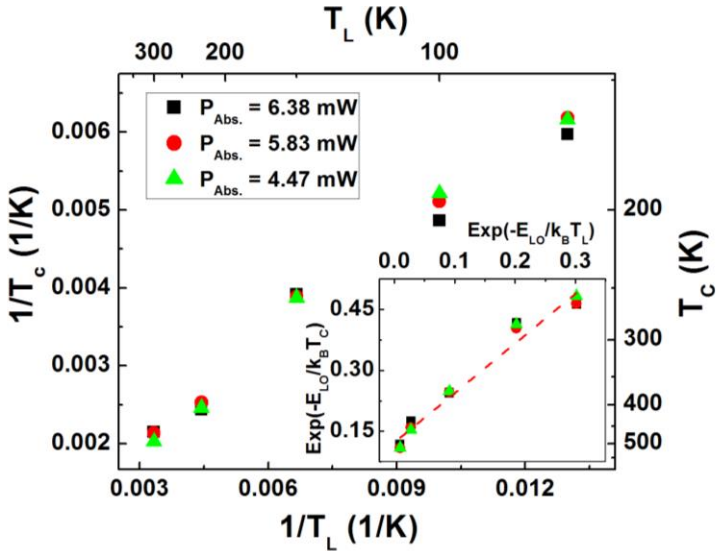

Figure S7.1: Reciprocal of the carrier temperature (1/T_C) versus lattice temperature reciprocal (1/T_L) for the process InAs/AlAs$_{0.14}$Sb$_{0.86}$ MQW at three different powers: high (solid black squares), medium (solid red circles), and low (solid green triangles). The inset shows a plot of the exponential components from Equation (2). Both the main figure and inset utilize Equation (2) to extract τ_{eff} the effective LO phonon scattering rate.

[1] V. R. Whiteside et al. *Manuscript is submitted for publication.*
[2] Kresse, G.; Furthmu ̈ller, *J. Phys. Rev. B* 1996, 54, 11169–11186.
[3] Perdew, J. P.; Burke, K.; Ernzerhof, M. *Phys. Rev. Lett.* 1996, 77, 3865–3868.
[4] Blochl, P. E. *Phys. Rev. B* 1994, 50, 17953–17979.
[5] Kresse, G.; Joubert, D. *Phys. Rev. B* 1999, 59, 1758–1775.
[6] Togo, A.; Tanaka, I. *Scr. Mater*. 2015, 108, 1-5.
[7] Gonze, X.; Lee, C., *Phys. Rev. B* 1997, 55, 10355
[8] Le Bris, A., et al. *Energy & Environmental Science* 5.3 (2012): 6225-6232.
[9] Ridley, B. K. *Journal of Physics C: Solid State Physics* 15.28 (1982): 5899.
[10] Balkan, N., et al. *Journal of Physics: Condensed Matter*14.13 (2002): 3457.
[11] Esmaielpour, Hamidreza, et al. *Progress in Photovoltaics: Research and Applications* 24.5 (2016): 591-599.
[12] Tang, J., et al. *Applied Physics Letters* 106.6 (2015): 061902.
